# Retraction Note to: Childhood nosocomial viral acute respiratory tract infections in teaching hospital Anuradhapura, Sri Lanka

**DOI:** 10.1186/s13104-021-05676-8

**Published:** 2021-07-06

**Authors:** Jayaweera Arachchige Asela Sampath Jayaweera, Mohammed Reyes

**Affiliations:** 1 Department of Microbiology, Faculty of Medicine and Allied Sciences, Rajarata University of Sri Lanka, Saliyapura, 50008 Sri Lanka; 2 Department of Pediatrics, Faculty of Medicine and Allied Sciences, Rajarata University of Sri Lanka, Saliyapura, 50008 Sri Lanka

## Retraction to: BMC Res Notes (2019) 12:581 10.1186/s13104-019-4624-2

The Editor has retracted this article. Following publication, concerns were raised that this study violated the approved protocol for this study. The ethical review committee of the University of Peradeniya confirmed that the approval as listed in the article does not cover the research as described in the article, specifically:

- The ethics approval was granted to include 572 patients, but the study population in the article contains data of 852 patients.
- The article includes follow up data regarding mortality rates that were not covered by the ethics approval.

The authors have also stated that the recruitment period as reported in the article (March 2015 to August 2016) is not correct and should be June 2013 to November 2014. Additionally, the authors have stated that they have collected data at two hospitals, though the article only lists one hospital. The editor therefore no longer has confidence in the reliability of the data reported in the article.

The authors don't agree with this retraction.

